# Efficacy and safety of mechanical transvenous lead extraction: median follow-up analysis and development of an experimental model for predicting survival post-extraction

**DOI:** 10.1186/s43044-025-00617-3

**Published:** 2025-02-25

**Authors:** Shima Nasri, Sahar Samimi, Masoud Eslami, Khashayar Hematpour, Morteza Eslami, Hirad Yarmohammadi, Reza Mollazadeh, Mehrzad Rahmanian

**Affiliations:** 1 Imam Khomeini Hospital Complex, Tehran University of Medical Sciences, Islamic Republic of Iran Tehran,; 2 McGovern Medical School, University of Texas, Houston, USA; 3 Columbia University Vagelos College of Physicians and Surgeons, New York, USA

**Keywords:** Transvenous lead extraction, Cardiac implantable electronic device, Device erosion, Endocarditis, Device infection, Lead removal, Pocket infection

## Abstract

**Background:**

Cardiac implantable electronic device (CIED) implantation is on the rise, accompanied by an increase in its inevitable complications such as different types of CIED infections that require further therapy and potential device extraction. Ensuring efficacy and safety remains paramount in transvenous lead extraction (TLE), given the complex nature of the procedure. The purpose of this study is to assess the outcomes of relatively low-cost mechanical TLE, including mid-term clinical follow-up, and to develop a predictive model for post-TLE survival. This study included all consecutive patients admitted for TLE at two tertiary medical centers between 2016 and 2021. Baseline characteristics, TLE procedure details complications occurring during and/or after the procedure and follow-up outcomes were collected.

**Results:**

During the 5-year period, 100 consecutive patients underwent TLE. The mean age of the subjects was 61 ± 3 years. The average time from lead implantation to TLE was 69.34 ± 9.36 months, with a total of 216 leads extracted. The most common indication for TLE was infection observed in 87% of subjects with pocket infection seen in the majority (84%). Complete clinical success was achieved in 98% of patients, with major complications occurred in 5% of cases and only one case of peri-procedural death. Proposed experimental model showed that near 50% of the patients will live less than 73.29 months.

**Conclusion:**

TLE demonstrated a high level of safety with low mortality and morbidity rates. Using low cost widely available mechanical tools is useful for treating CIED-related infections.

## Background

CIED implantation has increased worldwide largely due to the expansion of its indications, longer lifespan of patients with cardiovascular disease and improved access to healthcare [1]. These devices have improved morbidity and mortality in patients with a wide range of cardiovascular diseases [2]. Evidently this development is accompanied by complications such as local and systemic infections, lead dysfunction and many more which necessitate lead extraction [3]. The most widely accepted indication for lead extraction is infection. Bacteria can colonize all surfaces of CIED, rendering it impossible to eradicate infection solely with antibiotic therapy, so removal of all CIED components is crucial for resolving the infection [4]. TLE, although being superior to surgical methods, is considered a complex procedure that is still under-utilized in many countries [5]. Our goal was to report the safety, efficacy and outcomes of TLE in patients referred to two tertiary centers for TLE with mechanical tools from 2016 to 2021 and their follow-up and propose an experimental model for survival in these patients.

## Methods

### Study population

We conducted a retrospective study of involving all patients who underwent TLE at IKHC and Pars general hospital in Tehran, Iran, between 2016 and March 2021. Following approval from the local ethics committee (Tehran University of Medical Sciences), with the ethical code IR.TUMS.IKHC.REC.1399.212. Informed consent was obtained from all patients entered this study. The authors state that they have followed the principles outlined in the Declaration of Helsinki for all human or animal experimental investigations. Meanwhile, informed written consent has been obtained from the participants participated in the study. Clinical trial number: Not applicable.

### Data collection

Baseline characteristics including demographic features, comorbidities and relevant laboratory data including microorganism cultures (i.e., tissue, lead and blood), complete blood count, erythrocyte sedimentation rate and CRP were collected from patients’ charts. The initial indication for device implantation, device type and the duration until TLE, indication for TLE, number of removed leads, lead fixation mechanism and clinical outcome were also obtained.

### Definitions and procedure details

**Fig. 1:**
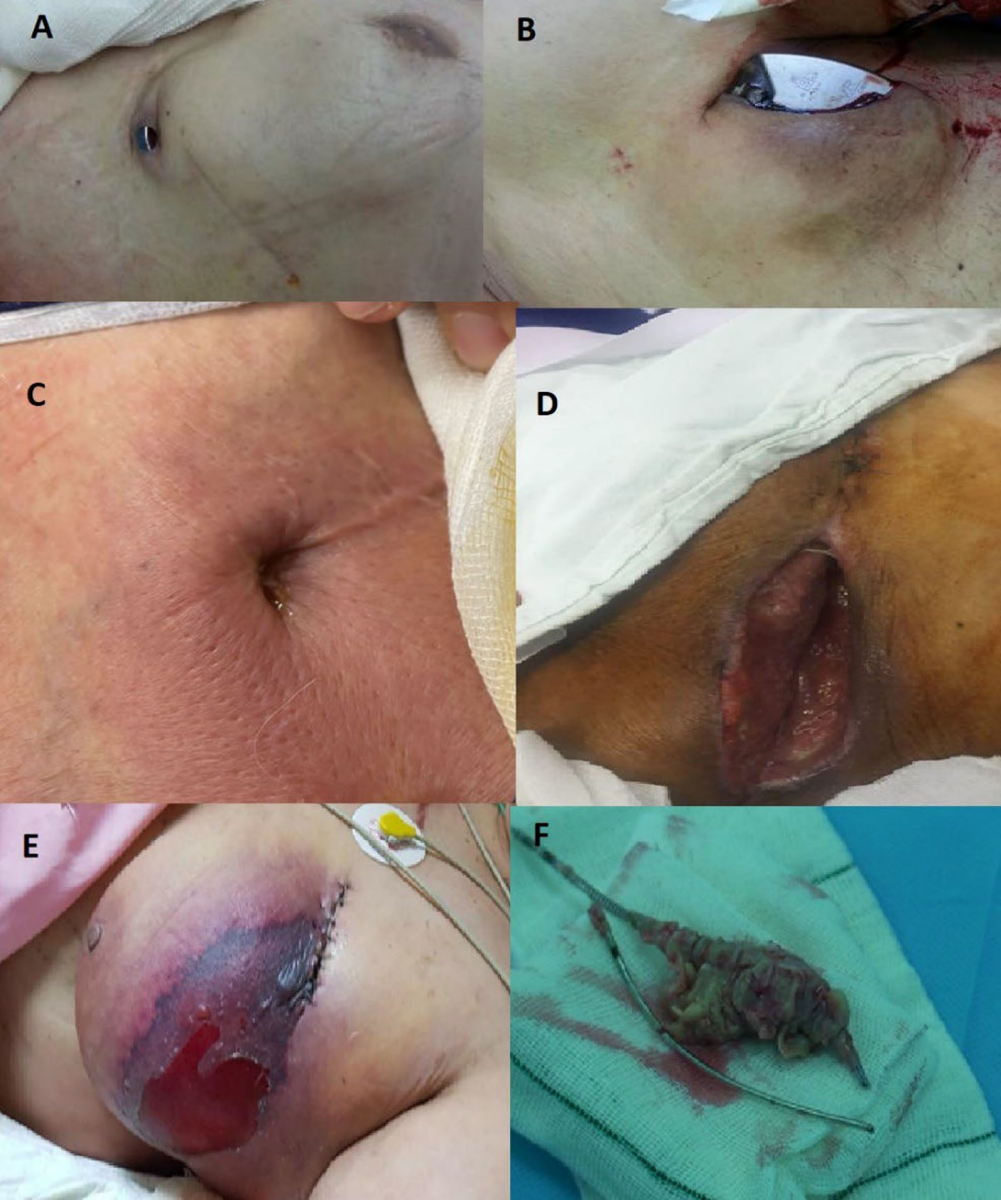
Different images of CIED infection: **A**: lead erosion, **B**: generator erosion, **C**: fistulous formation, **D**: wound dehiscence due to recurrent pocket revision, **E**: breast hematoma and necrosis and **F**: lead vegetation removed surgically

Indications for TLE were categorized as either infection or non-infection causes. Infection causes for TLE were further classified as local (i.e., pocket infection and/or lead/generator erosion) and systematic (i.e., bacteremia and/or lead vegetation or valve endocarditis) (Fig. 1) [6]. Non-infection causes for TLE were lead dysfunction, lead-related complications (e.g., thromboembolic events, superior vena cava syndrome, arrhythmias, perforation and lead–lead interaction), abandoned non-functional lead and venous access issues (e.g., stenosis) [7].

**Fig. 2:**
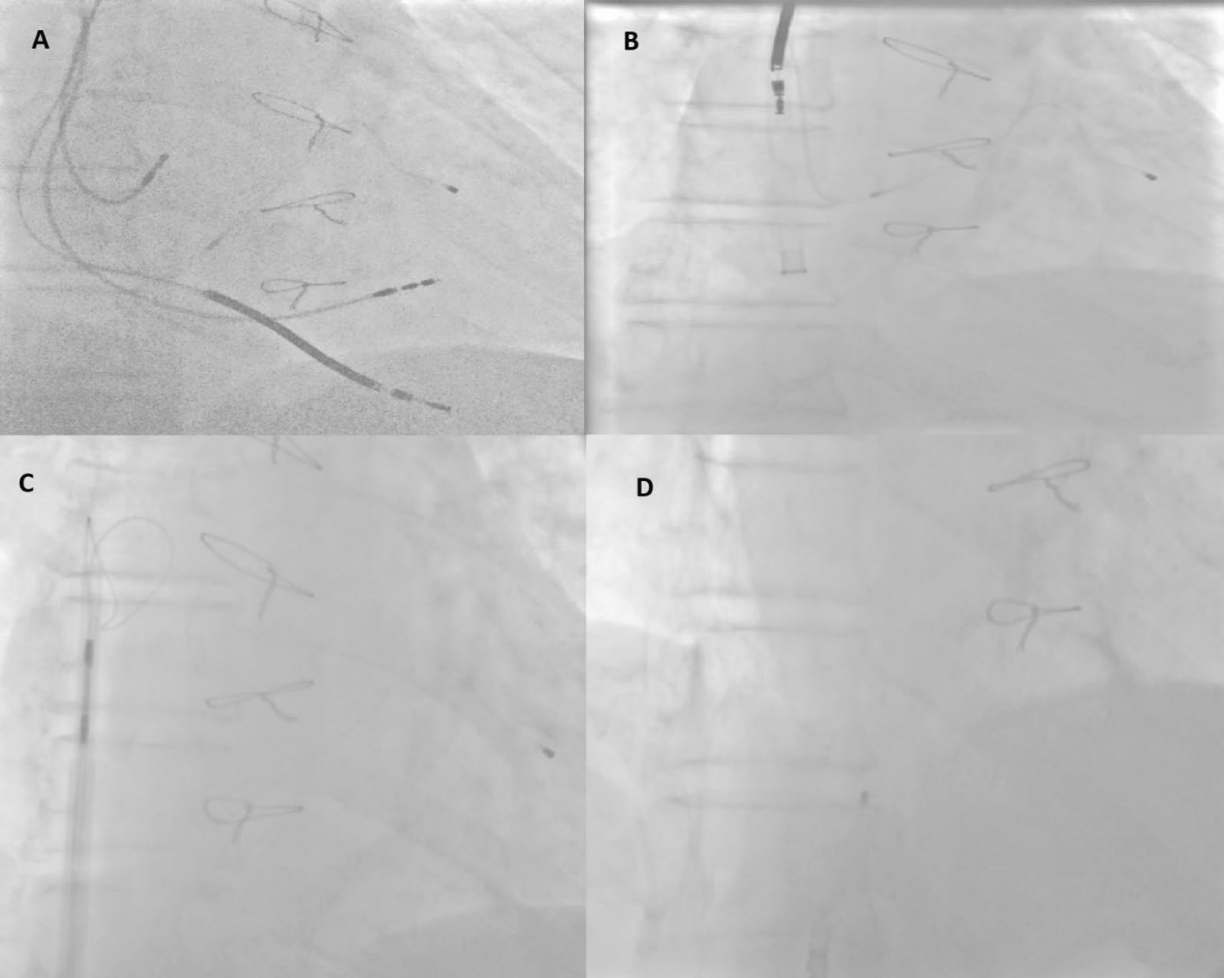
Example of stepwise approach: **A**: TLE in a patient with four leads in heart (right atrium, right ventricle pacing, high voltage and coronary sinus leads). **B**: Right atrium, right ventricle pacing and high-voltage leads could be extracted with locking stylet and hand-powered sheath. **C**: Coronary sinus lead was torn away during traction, countertraction and sheath advancement, so femoral approach using large deflectable sheath (Agilis™ NxT (Abbott, Chicago, IL, USA)) and Eye snare (Cook Medical LLC, Bloomington, IN, USA). D: Final result

All procedures were performed by expert electrophysiologists in catheterization laboratory with cardiac surgery back-up in hospital or on site (Hybrid room). TLE was approached in a stepwise manner: Unscrewing via standard stylets and simple manual traction via the initial entry site was used to extract the leads. If unsuccessful, assisted manual traction with a locking stylet and mechanical dilator sheath (Extor sheath, Biotronik, Berlin, Germany or Cook Medical LLC, Bloomington, IN, USA) and simultaneous traction–countertraction was utilized. Under circumstances that mechanical dilator sheaths fail, hand-powered mechanical Evolution RL controlled-rotation dilator sheath (Cook Medical LLC, Bloomington, IN, USA) was used in an attempt to break the fibrous adhesions and help disconnect lead-to-endocardial and lead-to-lead adhesions. If all else failed, a femoral approach was used to retrieve the leads (via conventional snares and Eye snare (Cook Medical LLC, Bloomington, IN, USA)) (Fig. 2).

Complete procedural success was defined as the removal of all targeted materials and leads. Death, cardiac or pulmonary arrest, vascular laceration, cardiac avulsion, cerebrovascular event, severe pericardial effusion requiring pericardiocentesis or surgical repair and pulmonary embolism requiring thrombolytics were considered as major complications [7]. Complications that did not meet these criteria were classified as minor complications.

### Follow-up

Lead, generator and tissue samples, as well as blood cultures, were obtained. Empirical antibiotic regimens were initiated pending the results of cultures and antibiogram sensitivity. Specific antibiotics were used and continued according to the type of microorganism. We follow up upon of all the patients’ statuses via phone call or the follow-up visits. The data on re-infection rates and mid-term survival were collected.

### Statistical analysis

Continuous variables were presented by mean ± standard deviation or as median (interquartile range) if not normally distributed. Frequencies and proportions were calculated for the categorical variables. Fisher exact tests were used to compare categorical variables. Continuous variables were compared across multiple groups using ANOVA test. P-values ≤ 0.05 were considered statistically significant. Kaplan–Meier and 3-parameter Weibull curves were constructed for survival model analyses. Kaplan–Meier curves are used for normally distributed parameters, and Weibull parameter-3 curves were used also for depiction of survival in experimental model. Statistical analysis was performed using IBM SPSS Statistics for Windows, version 20 (Armonk, NY: IBM Corp).

## Results

### Patients and leads characteristics

A total of 100 consecutive patients admitted for TLE were enrolled during a 5-year period from two referral medical centers. Patients’ average age was 61 ± 15, ranging from 16 to 89 years, and 20% of patients were female. Regarding past medical history and risk factors, 67% had HF. Only 7% of patients reported no conventional cardiovascular risk factors, and 80% had at least two conventional risk factors (diabetes mellitus, hypertension, hyperlipidemia and cigarette smoking) (Table 1). In their past drug history, 75% of the patients reported using antiplatelets, 21% anticoagulants and 2% corticosteroids. Forty-four percent of the patients had been treated with oral or intravenous antibiotics prior to referral to our centers for TLE. Patients’ laboratory data showed a mean hemoglobin and CRP of 11.98 g/dl and 31.79 mg/lit, respectively. Additionally, 29% of patients had a positive procalcitonin test (above 0.5 ng/ml) prior to TLE (Table 1).

**Table 1:** Baseline characteristics of the patients (number #100)

Demographic	Mean value/percentage
Mean age (years ± SD)	61.38 ± 14.5
Female	20
Heart failure	67
Ischemic heart disease	50
Chronic kidney disease	31
Diabetes mellitus	31
Previous cardiac surgery	28
Chronic obstructive pulmonary disease	6
Cerebrovascular disease	5
Anemia	64

Drug history	Percentage
Antiplatelets	75
Antibiotics	44
Anticoagulants	21
Corticosteroids	2

Laboratory data	Mean (95% CI)
White blood cells (× 10^3^/mm^3^)	7.82 (7.2–8.4)
Platelets (× 10^3^/mm^3^)	213.52 (200–226)
Hemoglobin (g/dl)	11.98 (11.5–12.41)
Mean INR	1.26 (1.19–1.33)
Erythrocyte sedimentation rate (mm/hr)	35.26 (29–41)
C-reactive protein (mg/L)	31.79 (23–40)

INR: international normalized ratio

The proportion of type of CIED among patients was as follows: 23, 21 and 30 percent received single-, dual- and three-chamber ICD. Single- and dual-chamber PPM accounted for 6 and 20 percent of the CIEDs, respectively. Twenty-four, 34, 28 and 14 percent of the patients had 1, 2, 3 and 4 leads, respectively (on average 2.16 leads per patient). Ninety-one percent of the leads had active fixation mechanism (Table 2).

**Table 2:** Characteristics of the devices and leads extracted

Device type	Percentage
Dual-chamber PPM	20
Single-chamber PPM	6
Single-chamber ICD	23
Dual-chamber ICD	21
CRT-D	30

Lead type	Number (Percentage)
Pacemaker	127 (63)
High voltage	74 (37)

Indication of implantation	Percentage*/months* ± *SD*
Primary prevention ICD	45
Secondary prevention ICD	34
Bradyarrhythmia	21
Mean lead indwelling time	69.34 ± 50.34

Device manufacturer	Percentage
Medtronic	59
Saint Jude	25
Biotronik	8
Boston scientific	7
Ela-Sorin	1

Indications for transvenous lead extraction	Percentage
Infectious	87
Pocket infection	84
Lead vegetation	37
Bacteremia	31
Non-infectious	13
Lead malfunction	8
Venous access complications	3
Lead-related complications	2

Number of leads removed per patient	Percentage
1	29
2	37
3	23
4	11

Positive culture	Percentage
Lead	49
Blood	29
Tissue	49

PPM: Permanent pacemaker, ICD: Implantable cardiac defibrillator and CRT-D: Cardiac resynchronization therapy-defibrillator

The average time from device implantation to TLE was 69.34 ± 50.34 months (ranging from 4 to 250 months), and TLE was clinically successful in 98% of patients according to the definition of success in consensus statement [6]. The number of leads extracted for each patient was 1, 2, 3 and 4 leads in 29%, 37%, 23% and 11% of patients, respectively (average 2.16 leads per patient).

The most common indication for TLE was infection (87%). In the infection group, 84% had evidence in favor of pocket infection, 37% had lead vegetation or endocarditis and 31% had bacteremia (as is evident some patients had more than one site of infection). Thirteen percent of the patients underwent TLE due to other causes: 8% lead malfunction, 3% due to venous obstruction and 2% due to lead complications (perforation, oversensing and/or inappropriate shocks). Lead and wound cultures were positive in 49%, while blood cultures were positive in 29% of the patients. The most common microorganism cultured was CoNS in 28% of patients, followed by MRSA in 24%. However, regarding blood culture results, MRSA was the most common microorganism and then CoNS at 24% and 21%, respectively (Table 2).

### Major and minor complications of TLE procedure

Major complications occurred in five percent of the patients with just one intra-procedure death. The deceased patient was a case of CIED-related pocket infection and infective endocarditis that was referred to our center after failure of 4 weeks of broad-spectrum intravenous antibiotic therapy. Transthoracic and trans-esophageal echocardiography showed a relatively large vegetation on the tricuspid valve measuring 1.7 × 2.5 cm. Concomitant patent foramen ovale was also present. She was septic despite antibiotic treatment, and she was found to not be a surgical candidate. After termination of TLE procedure and anesthesia, the patient was not responsive. Pupils were non-reactive. Imaging showed occlusion of left-sided common carotid artery with infarction of ipsilateral of brain. The patient died 2 h later. Detailed results of TLE complications are brought in Table 3.

**Table 3:** Major and minor complications of TLE

Major complications	Percentage
Intra-procedural death	1
Cardiac arrest	1
Pulmonary arrest	1
Vascular laceration	1
Cardiac avulsion	0
Cerebrovascular event	1
Severe PE^1^	0
Severe PTE^2^	0
Total	5

Minor complications	Percentage
Vascular repair	0
PE	0
Hematoma	10
Pneumothorax	2
Bleeding	7
PTE/DVT	3
Reinfection	4
TLE unsuccess	2

^1^Severe PE: Pericardial effusion requiring pericardiocentesis

^2^Severe PTE: Pulmonary thromboembolism requiring thrombolytics

PE: Pericardial effusion, PTE: Pulmonary thromboembolism, DVT: Deep venous thrombosis, and SD: Standard deviation

### Median term follow-up

**Fig. 3:**
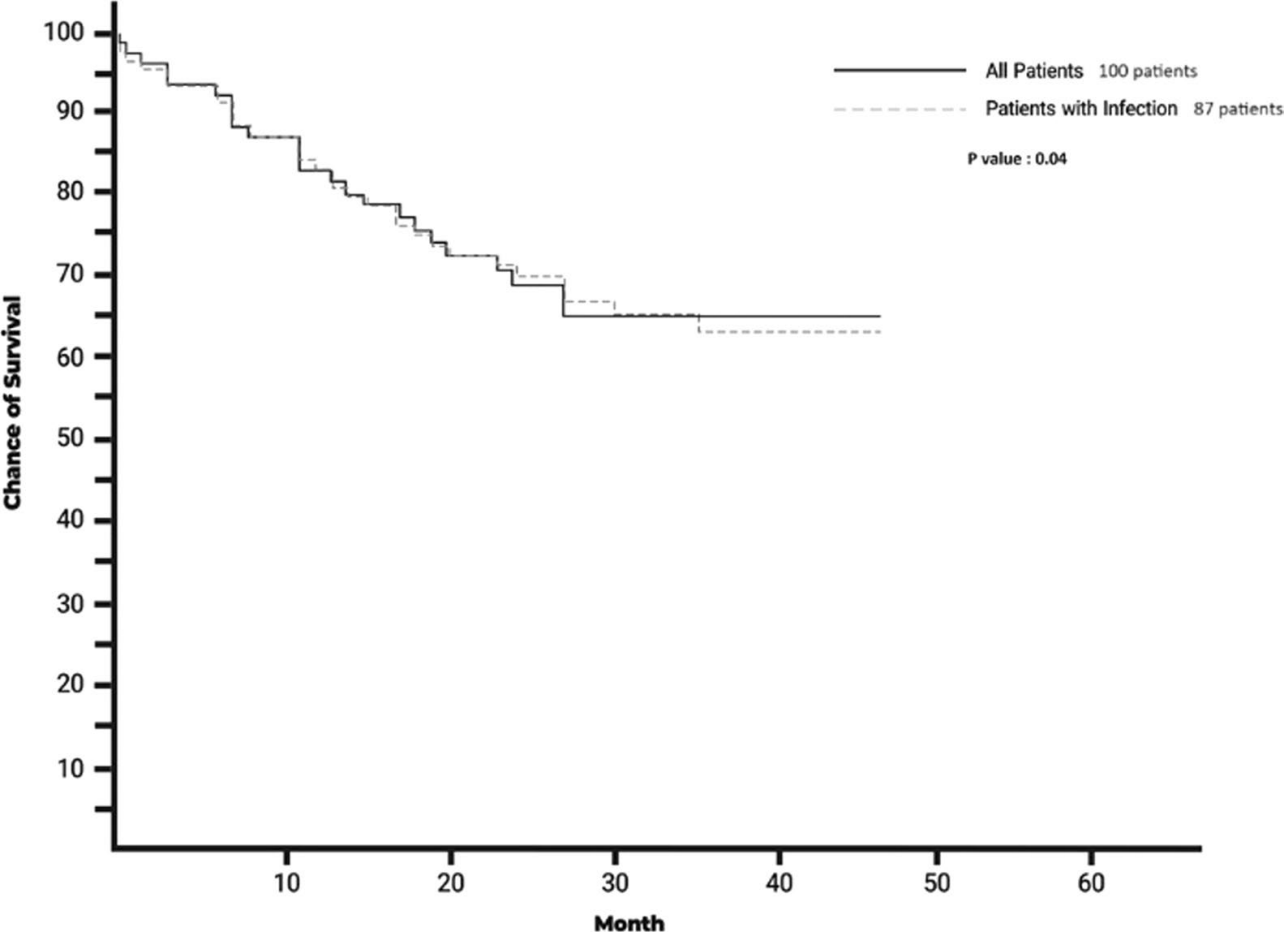
Survival Kaplan–Meier curve (months) of all patients (black line) and patients with infection (dashed line) undergone TLE

Patients were followed on average for 33.61 months. Outpatient clinic visits or trans-telephonic queries were used for follow-up and survival. Time from TLE to death was longer for patients without CIED infection at 26.50 ± 16.70 months, compared to 12.26 ± 8.19 months for subjects with infection (*p*-value = 0.04) (Fig. 3 Kaplan–Meier curve).

## Experimental model of survival in follow-up

**Fig. 4:**
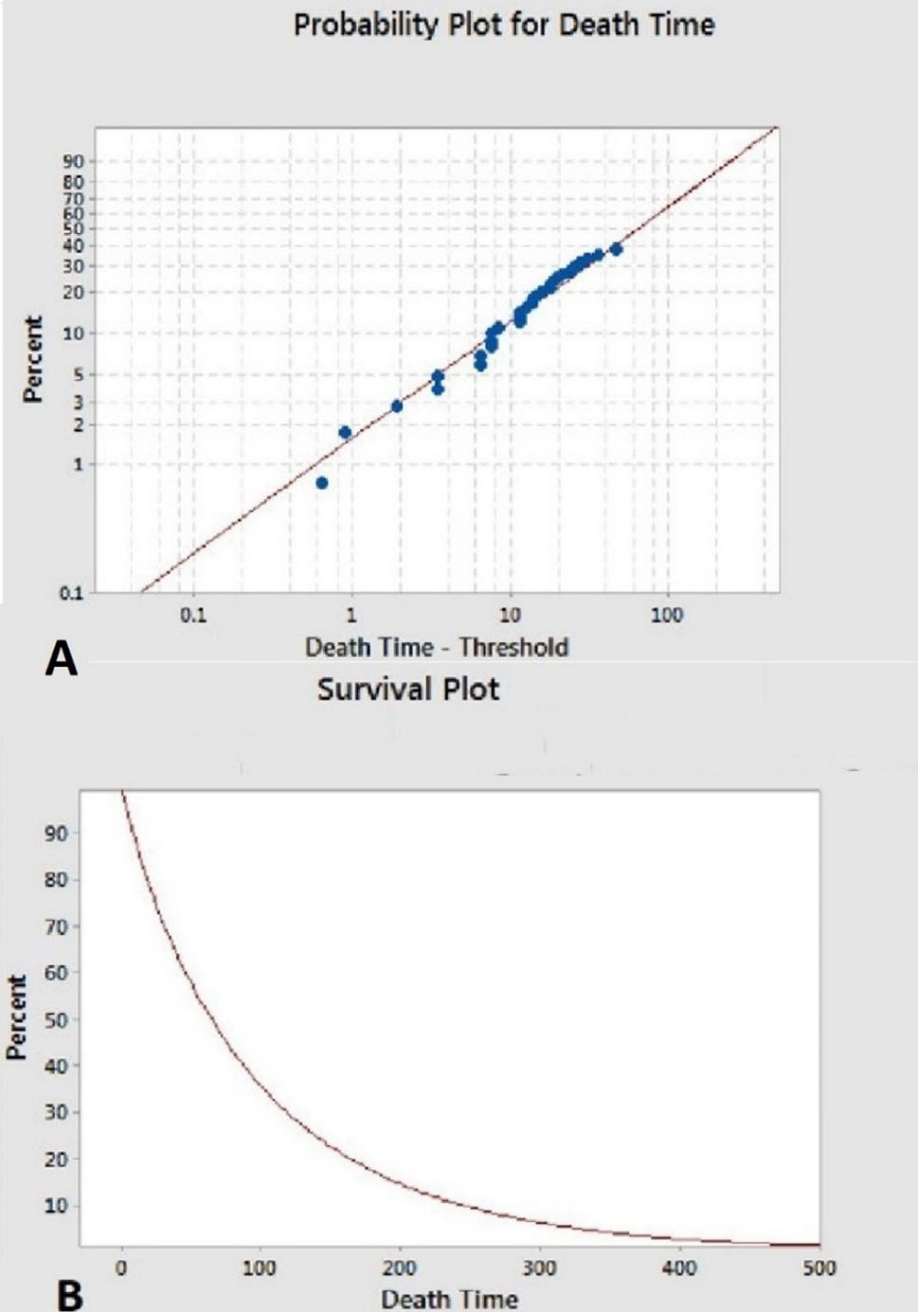
Figure 4A shows that the mean survival time after TLE is less than 100 months and about half of patients will live less than 63.8 months (median time). Based on Fig. 4B, less than 20% of patients after TLE will live probably longer than 200 months. This means that about 80% of patients are expected to die within 17–18 years of TLE

Figure 4 shows the experimental model diagrams for the survival (according to months) after TLE for all indications. Figure 4A shows that the mean survival time after TLE is about 100 months, and about half of patients will live less than 63.8 months (median time). Based on Fig. 4B, less than 20% of patients after TLE will live longer than 200 months. This means that about 80% of patients are expected to die within 17–18 years after TLE.

## Discussion

The current study aimed to investigate the outcome of mechanical TLE in patients referred to two tertiaries medical centers in Tehran, Iran. To date, this is the first report of the multicenter study of safety and efficacy of mechanical TLE in our country. Finally, in this study, we presented an experimental model for the survival time after TLE.

Patients in this study had similar demographics to the previous reports, with mostly male patients and an average age of 61.38 years. Additionally, like the previous studies, HF (67%), ischemia heart disease (50%), chronic kidney disease (31%) (one patient had end-stage renal disease and on regular hemodialysis) and diabetes (31%) were common comorbidities seen in patients [5, 8]. Most patients had multiple comorbidities, which as expected increases with age.

Similar to the previous studies, infection, specifically pocket infection, was the most common indication for TLE, although much higher than ELECTRa registry (87% compared to 54%). This finding was also affected by the fact that health insurance companies in our country only reimburse TLE costs indicated due to CIED infection.

The clinical success rate of 98% in the present study was in-line with the previous large studies ranging from 96.7% to 99.4% clinical success [5, 9]. Earlier studies (PLEXES and LExICon) that incorporate laser TLE reports significant higher success rates especially compared to simple dilator sheaths (94% vs. 64%) [10, 11], but with emergence of hand-powered bidirectional rotational sheath, this difference was minimized to 1–2% (PROMET and RELEASE studies) [9, 12].

In the ELECTRa registry, 41 (1.2%) major complications, and in a comprehensive study by Brunner et al., 54 (1.8%) major complications occurred [5, 13, 14]. In our study, we had higher major complication rate (5%). We assume that it is due to more complex nature of the procedure. TLE program was started in our country in 2016, before that physician in charge performed several times of pocket revisions by themselves or cardiothoracic surgeons. Meanwhile as previously stated, higher percentage of our patients underwent TLE due to infection and higher rate of complications in TLE procedures among patients with infection were anticipated and observed in the previous studies [5, 8]. Another fact is the delay in referral of the patients to our canters from far away local hospitals, this is as previously addressed is associated with increased in-hospital mortality and major adverse events especially in patients with systemic infection [15]. Fortunately, in this study, we had only one intra-procedural death (1%) that was comparable to the previous literature with procedural mortality up to 0.6% [8]. Other probable reason for higher rate of complications in our study is that near 70 percent of procedures included ICD leads that majority were dual-coil ICD leads (including superior vena cava (SVC) coil). Adhesion of SVC coil to endothelium and higher chance of SVC rupture in case of trauma to vessel wall in case of inadvertent force/push is a well-known challenge in TLE [16, 17].

The average number of leads extracted for each patient were 2.16 which is a bit higher than the ELECTRa study with an average of 1.8 leads [5]. This is probably due to the underdeveloped TLE practice in our country, which means most electrophysiologists implanted new leads in the event of lead dysfunction without removing the prior leads. Still, this is current in our country. In our country (like many developing countries), TLE is limited to few centers and actually it is not fully re-imbursed [18]. Therefore, upon referral of the patient for TLE, higher number of leads were removed per patient.

Follow-up of our patients revealed that TLE due to infection was significantly associated with higher long-term mortality, which was compatible with the previous studies [8, 19]. Deckx et al. who investigated mortality after TLE in a single-center retrospective cohort revealed that systemic infection significantly increased 30-day, 1-year and long-term mortality [20]. Maytin et al. witnessed an increase from 2.1% to 10% in 30-day mortality in patients with CIED systemic infection [21]. Gould et al. similarly reported a significantly higher 30-day all-cause mortality and all-cause major complications in patients undergoing TLE in the infective group. Their study also stated that patients with systemic infections were at a higher 30-day mortality risk compared to local infections [22].

In the present study, patients without CIED infection lived more than twice as longer after lead extraction compared to subjects with infection (26.5 vs. 12.2 months P-value < 0.05). Due to low number of patients, it was not plausible to perform subgroup analysis to evaluate different types of infection (local (lead or generator erosion, pocket abscess) or systemic (bacteremia, lead or valve vegetation)).

We proposed experimental model for long term (more than 10 years) after TLE in both groups of patients with and without infection etiology. This figure is based on Weibull statistical model if similar survival rate remains post-TLE. Patients with CIED infection undergoing TLE already had multiple comorbidities (heart failure, diabetes mellitus and chronic kidney disease) which already poses them to higher baseline mortality rates [2]. So long-term survival in experimental model is expectable and would be much lower than general population.

## Conclusion

This study confirms the safety and appropriateness of using mechanical TLE with limited major and minor complications and acceptable risk–benefit ratios. This is also confirmed in the newer studies by an Italian group with good expertise in TLE using mechanical sheaths [23–25]. Although TLE for CIED-related systemic infection has been demonstrated to be associated with a significantly higher mortality rate.

## Limitations

The findings of our study are mainly limited by the two-center design of the study and small sample size. Secondly, due to the observational nature of the study, it was susceptible to unknown bias and confounders. Due to the retrospective design of study, unmeasured significant confounders could not be excluded. The final limitation was the presence of the COVID-19 pandemic, and IKHC being the main center to treat affected patients. This partially hindered the referral of patients for TLE during this time and limited the study population size. Noteworthy that due to small sample size, subgroup analysis could not be performed.

## Data Availability

No datasets were generated or analyzed during the current study.

## Abbreviations

CIED: Cardiac implantable electronic device
CONS: Coagulase-negative staphylococci
CRP: C-reactive protein
ICD: Implantable cardioverter defibrillator
HF: Heart failure
MRSA: Methicillin-resistant staphylococcus aureus
PPM: Permanent pacemakers
TLE: Transvenous lead extraction
